# Physical, Rheological, and Morphological Properties of Asphalt Reinforced by Basalt Fiber and Lignin Fiber

**DOI:** 10.3390/ma13112520

**Published:** 2020-06-01

**Authors:** Changjiang Kou, Xing Wu, Peng Xiao, Yang Liu, Zhengguang Wu

**Affiliations:** 1 College of Civil Science and Engineering, Yangzhou University, Yangzhou 225127, China; wuxingyzu@126.com (X.W.); xpyzu_mail@163.com (P.X.); zgwu@yzu.edu.cn (Z.W.); 2 Centre for Pavement and Transportation Technology, University of Waterloo, Waterloo, ON N2L 3G1, Canada; frank.liu@uwaterloo.ca

**Keywords:** mixed fiber-reinforced asphalt, fiber mix ratio, basalt fiber, lignin fiber, comprehensive performance

## Abstract

Studies show that each kind of fiber has its own advantages in improving the properties of asphalt binders. However, there are very limited research studies about mixed fiber-reinforced asphalt (MFRA). In this study, two kinds of fibers, basalt fiber (BF) and lignin fiber (LF), were selected to reinforce SBS (styrene–butadiene–styrene triblock copolymer)-modified asphalt, which is now widely used in pavement engineering. MFRA samples with different fiber mix ratios (FMRs) were prepared for the tests of softening point, ductility, and rheological properties, the micromorphology of which was studied by using scanning electron microscope (SEM). The oil (asphalt) absorption rates of mixed fibers with different FMRs were also tested. The results show that the properties of MFRA were affected by the physical and chemical properties of fibers. Basalt fiber can better strengthen the physical properties of MFRA, while lignin fiber is good for improving the rheological properties, and the oil absorption rate of lignin fiber is higher than that of basalt fiber. Furthermore, the best FMR calculated by the efficacy coefficient method (ECM) was recommended as 1:2 (BF:LF). An interface layer between the fiber and asphalt was observed from the micro images, proving that the fibers bond well with the asphalt. Generally, mixing BF and LF together into SBS-modified asphalt could make full use of the advantages of different fibers and reinforce the comprehensive performance of MFRA better.

## 1. Introduction

With the rapid development of the modern economy, more and more fibers are being used in scientific research and engineering practice. Using fibers as reinforcements can often have better economic and performance advantages in extending the service life of matrix materials [1,2,3]. In the field of road engineering, many scholars have done a lot of research on the asphalt mixtures reinforced by different fibers, and found that basalt fiber (BF) and lignin fiber (LF) can significantly enhance the performance of asphalt and asphalt mixtures [4,5,6]. As a kind of composite material, the performance of asphalt mixture depends largely on its composition [7]. In asphalt mixtures, fibers are mainly combined with asphalt, and they together play a very important role in the asphalt mixtures. There are some evidences showing that the impact of fiber on asphalt has a good correlation with the impact on asphalt mixture [8,9,10]. Scholars around the world have begun to pay attention to the theoretical study of the performance of fiber-reinforced asphalt mortar [11,12,13].

Here are some details of several studies on the fiber-reinforced asphalt and asphalt mixtures reinforced by BF or LF. Cheng et al. [14] studied the effect of basalt fiber and diatomite on the performance of asphalt mixtures and found that the addition of basalt fiber is the main reason for improving the performance of asphalt mixture. Punya et al. [15] found that the addition of basalt fiber can reduce the penetration value of asphalt and increase the softening value. Wei et al. [16] studied the modification effect of basalt fiber on asphalt, and it was found that the rutting resistance property of SBS-modified asphalt increased significantly after basalt fiber was added. Wang et al. [17] found that basalt fiber could significantly reduce the damage degree of asphalt mixture by about 25% through the freeze–thaw cycle test. Fu et al. [18] used anti-rutting agent and lignin fiber to improve the performances of asphalt mixtures. After adopting these two additives, the high and low temperature and water stabilities were improved obviously. Xu et al. [3] investigated the reinforcing effects of lignin fiber and other three kinds of fibers on the asphalt mixtures and found that fibers can significantly improve the rutting resistance, fatigue life, and stiffness of the asphalt mixtures. Many studies have focused on the influence of single fiber on the performance of asphalt mortar or mixture or the comparison of the influences of multiple fibers on the performance of asphalt mortar or mixture. In fact, adding mixed fibers into the asphalt could make full use of the advantages of different fibers and get a better reinforcement effect on the asphalt. The addition of different fibers will have a synergic effect on the asphalt, making the mixed fiber-reinforced asphalt (MFRA) have all the advantages of the corresponding single-fiber reinforced asphalt binders. In the meantime, different fibers will form a more complex system in the MFRA, which will make it more stable. However, the study about the performance or the strengthening mechanism of mixed fiber-reinforced asphalt (MFRA) was ignored. 

Therefore, this paper considers adding BF and LF together into SBS (styrene–butadiene–styrene triblock copolymer) modified asphalt to study the performance and strengthening mechanism of mixed fiber-reinforced asphalt (MFRA). The MFRA samples were designed using several different fiber mix ratios (FMRs). Tests of softening point and ductility were chosen to study the high and low temperature physical properties of MFRA; a dynamic rheology test was chosen to study the rheological properties of MFRA; and the Scanning Electron Microscope (SEM) test was chosen to observe the micrograph of MFRA to explain the strengthening mechanism of MFRA. The conclusions of this study have certain meanings for the design of fiber-reinforced asphalt. As the influence of fiber on asphalt has a good correlation with the influence on asphalt mixture [8,9], this study is not only of great significance to guide the design of fiber-reinforced asphalt but also of great importance on the design of fiber-reinforced asphalt mixture.

## 2. Materials and Methods 

### 2.1. Materials

#### 2.1.1. Asphalt

In this paper, the SBS-modified asphalt was provided by Jiangsu Tiannuo Road Materials Technology Co., Ltd., Zhenjiang, China. The properties of it are listed in Table 1.

#### 2.1.2. Basalt Fiber and Lignin Fiber

**Figure 1:**
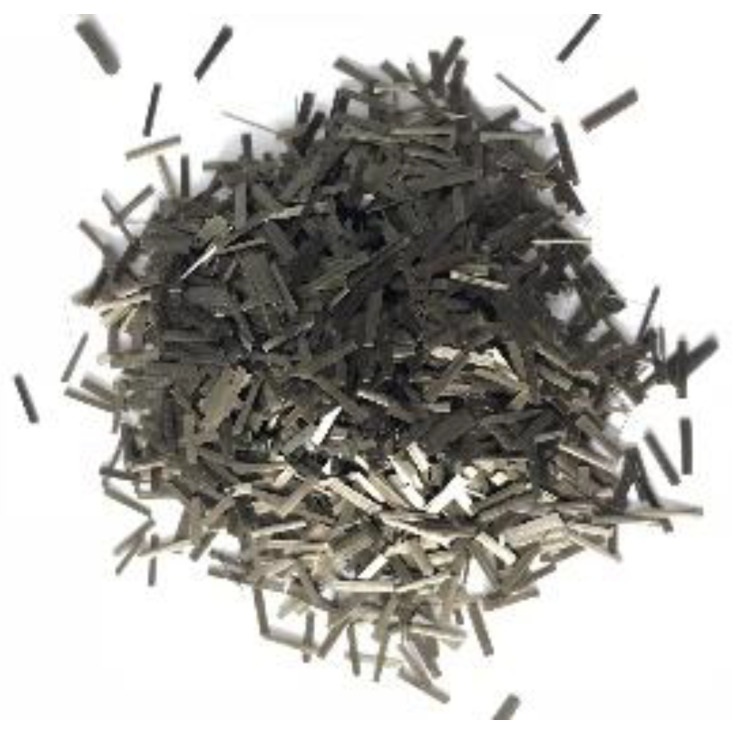
Macro image of basalt fiber.

Basalt fiber (BF) is produced from natural basalt at high temperature [19], and the manufacturing process is very environmentally friendly as there is no harmful by-product in its production. The performance of BF under high and low temperature is satisfactory, and it also presents promising properties regarding stability and thermal insulation. In this paper, the 6 mm chopped basalt fiber produced by Jiangsu Tianlong Basalt Continuous Fiber Co., Ltd., Yizheng, China, is used in the experiment. The macro image of basalt fiber is shown in Figure 1. It can be seen from the macro image that the color of basalt fiber is golden brown, and it separates from each other.

**Figure 2:**
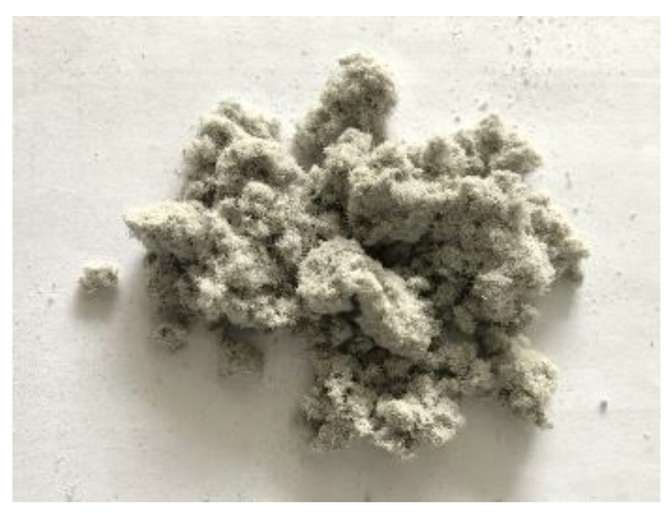
Macro image of lignin fiber.

Lignin fiber (LF) is made from natural wood. Various application requirements can be obtained via a series of chemical treatments to LF [20], during which the functional groups on the molecular structure of lignin changed. LF is flocculent, it tends to agglomerate and absorb moisture, so it is not suitable to be stacked for a long time in spite of its good chemical stability. The LF used in this paper is produced by The JRS company of Germany, Rosenberg, Germany. The macro image of it is shown in Figure 2. It can be seen from the macro image that the micro morphology of LF is quite different from that of BF. LF is flocculent and it is similar to cotton. Its color is gray and it is easy to be stuck together. 

The properties of basalt and lignin fibers given by the manufacturers are shown in Table 2. The PH value was tested by the aqueous extract method, and fibers were added into the aqueous solution to make the test samples. It can be concluded from Table 2 that the pH value and specific surface area of LF are larger than those of BF; the length–diameter ratio (LDR), heat resistance, and fracture strength of BF are larger than those of LF. The increase of the viscosity of fiber-reinforced asphalt depends on the content of fiber and the Einstein coefficient K_E_ [21]. The Einstein coefficient K_E_ is positively correlated with the length–diameter ratio and the viscosity of asphalt. Therefore, BF can better improve the viscosity of asphalt. The PH value of LF is larger than BF and they are all alkaline, which means that LF has a better chemical bonding with asphalt because asphalt is a weak acid material [22]. It is clear that these two fibers have their own advantages, which could further verify the feasibility of adding different fibers into asphalt to get a better performance of asphalt mortar.

### 2.2. Experimental Design

**Figure 3:**
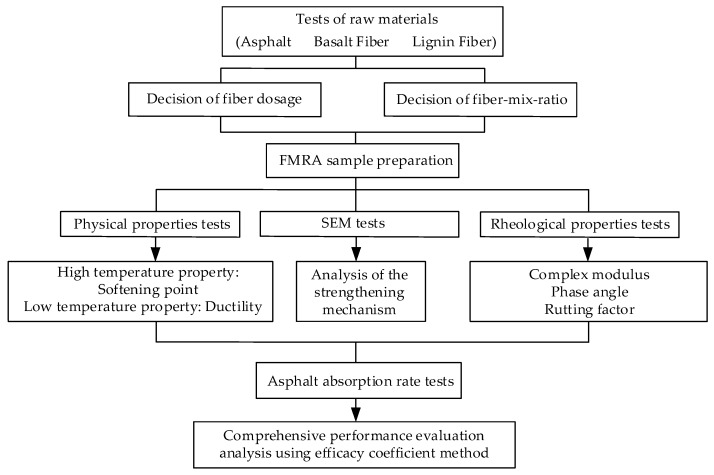
Flowchart of the experiments.

The flowchart of the experiments in this study is shown in Figure 3.

### 2.3. Test Methods

#### 2.3.1. Selection of Fiber Mix Ratio and Fiber Content

Fan et al. [23] conducted research on the performance of asphalt mixture reinforced by basalt fiber, and they found that the optimum content of fiber in the basalt fiber-reinforced asphalt mixture was 0.3% of the weight of asphalt mixture. However, in the study about fiber-reinforced asphalt, the fiber content should be expanded to ensure a more credible result. Zhang Min [24] took 3% of the weight asphalt as the recommended content of LF in the study of fiber-reinforced asphalt. Therefore, in this paper, 3% is selected as the total content of BF and LF in the SBS-modified asphalt to make different mixed fiber-reinforced asphalt (MFRA), and the different fiber mix ratios shown in Table 3 were set to study the influence of fiber mix ratio (FMR) on the performance of MFRA. FMR value means “Content of BF:Content of LF”.

#### 2.3.2. Physical Properties Tests

Softening point was chosen to represent the high-temperature property of MFRA, and ductility (5 °C, 5 cm/min) was chosen to represent the low-temperature property. The tests were conducted according to ASTM D36 [25] and ASTM D113 [26], and all the tests are conducted on three parallel samples. If the error between the value of each test and the average value is within 10%, the average value is taken as the final result. If not, all the tests will be conducted again.

#### 2.3.3. Rheological Properties Tests

**Figure 4:**
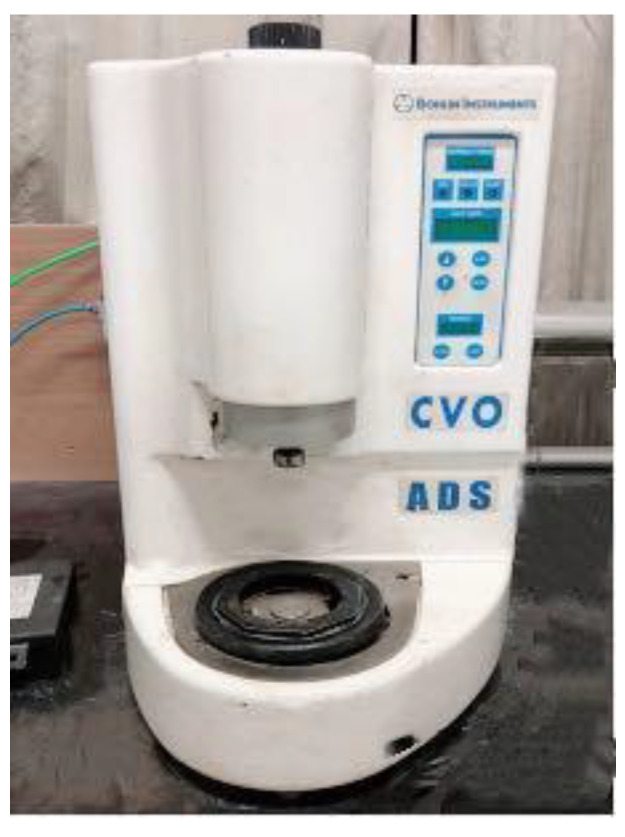
Dynamic shear rheometer.

**Figure 5:**
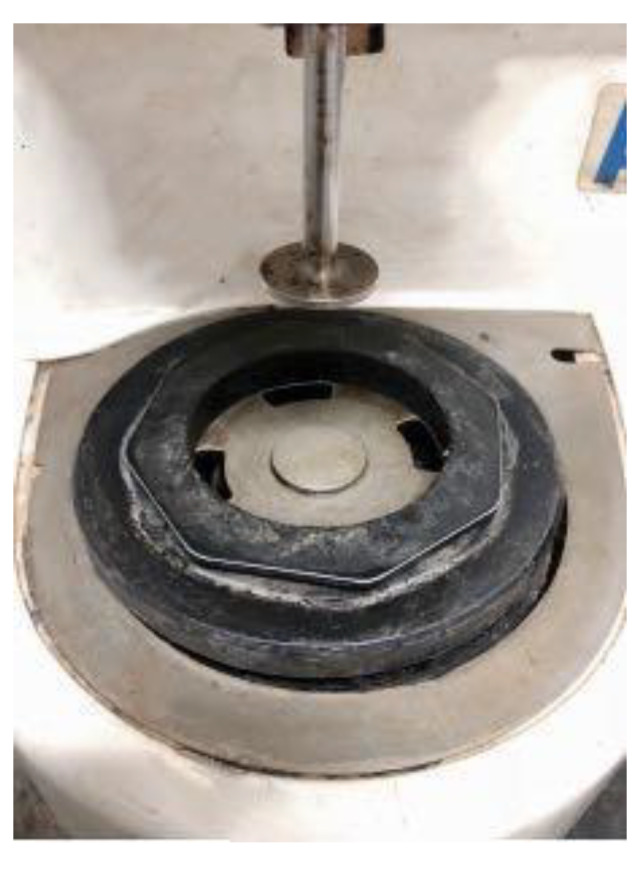
The parallel plates.

The dynamic shear rheometer used in this paper is produced by Malvern Instruments Co., Ltd., Malvern, UK, as is shown in Figure 4. According to ASTM D7175 [27], the diameters of the parallel plates (Figure 5) are 25 mm, and the gap value is set as 1000 μm. The strain-controlled loading mode is adopted in this paper, and the controlled strain was set as 1%. The test samples were placed between these two parallel plates. During the test, the lower plate remains fixed and the upper plate continuously swings back and forth around the central axis. It is obvious that the driving loads on asphalt pavement is dynamic, so the dynamic shear frequency is closely related to the traffic volume of the pavement. For example, high frequency can simulate heavy traffic or high speed, and low frequency can simulate light traffic. Therefore, in order to make the tests more similar to the actual situation, the complex modulus (G*), phase angle (δ), and rutting factor (G*/sin δ) of MFRA were tested at 60 °C with different test frequencies.

#### 2.3.4. Oil (Asphalt) Absorption Rate Tests

**Figure 6:**
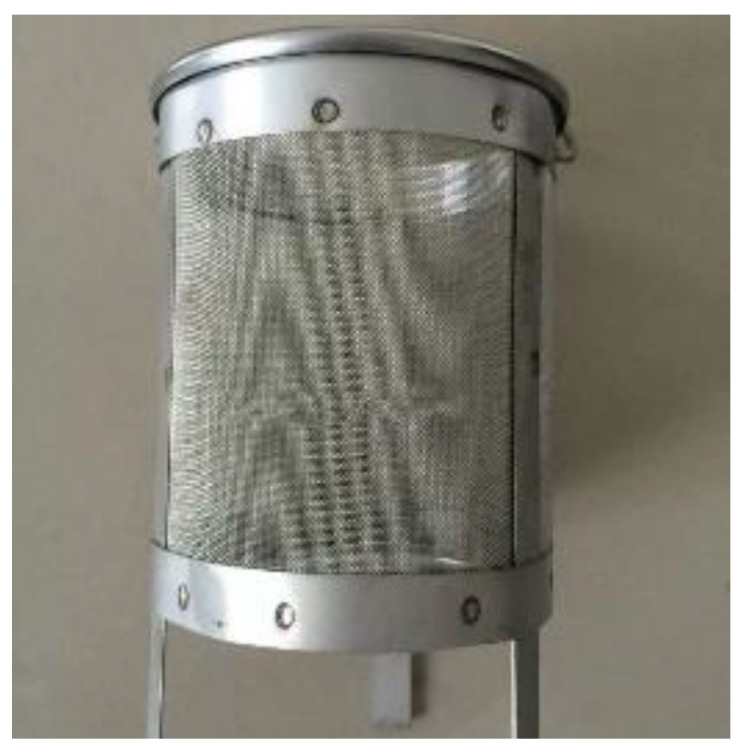
Basket used in the tests.

The surface of the fiber and the fiber itself can absorb a certain amount of asphalt binder in the asphalt mixture. The oil absorption rate can affect the properties of asphalt mixtures [28]. Within some range, the higher the absorption rate, the less likely the fiber-reinforced asphalt mixture will suffer from pavement distress such as oil-bleeding or ruts at high temperatures. Therefore, the asphalt absorption capacity of fibers is an important index to evaluate the performance of asphalt mortar or mixture. In this paper, the oil absorption rate of basalt fiber (BF) and lignin fiber (LF) is tested by basket leak test. The basket used in the test is shown in Figure 6; the diameter of the basket leaking hole is 0.315 mm, and the weight of the basket is 200 grams.

The steps of the test are as follows: (1) Place BF and LF separately into two cups, and put it in an oven (105 °C ± 5 °C) for 2 h. (2) Take BF and LF out according to the fiber mix ratio (FMR), making sure the total weight (*m*_1_) is 9 grams, and put it in a beaker. (3) Put the beaker on the tray balance and press the reset button. (4) Pour 300 grams of hot SBS asphalt into the beaker and stir it evenly with a glass rod. (5) Measure the weight of the clean basket and mark it as *m*_2_. (6) Put the basket on the top of a collection container and pour the fiber–asphalt mixture all into the baskets and be careful not to make the test sample drip or drain in the testing process (the diameter of the collection container is bigger than that of the basket). (7) Put the basket along with the collection container into an oven (165 °C) for 12 h until the weight of the basket is constant. (8) Measure the weight of the basket (with fibers and asphalt in it) and mark it as *m*_3_. The asphalt (oil) absorption rate (*OA*) is calculated according to Equation (1).

(1) $$OA=\frac{m_{3}-m_{2}-m_{1}}{m_{1}}$$

#### 2.3.5. SEM Tests

Scanning electron microscope (SEM) used in this paper is produced by Philips company Amsterdam, the Netherlands. Micro images of different kinds of mixed fiber-reinforced asphalt (MFRA) are taken in a vacuum condition, and the fiber mix ratios (FMRs) adopted in this test are 3:0, 1.5:1.5, and 0:3 because when the FMR is 2:1 or 1:2, the micro morphology is almost the same as the micro morphology when the FMR is 1.5:1.5. The purpose of this test is to explain the strengthening mechanism of the fibers when they are combining with the asphalt. 

**Figure 7:**
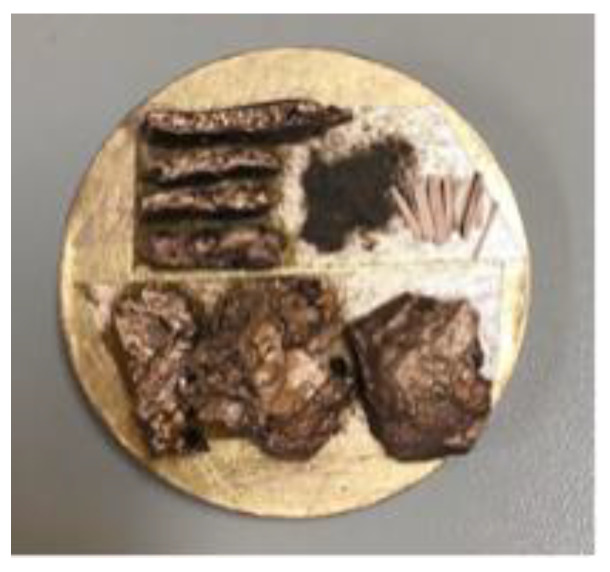
SEM test samples.

The steps of the sample preparation are as follows: (1) Prepare the MFRA sample in a mold. (2) Put it in a freezer and freeze it for 3 h. (3) Slice the sample with a clean blade. (4) Dry it in room temperature and make sure there is no water on the surface. (5) Plate gold in the vacuum coating machine (Figure 7). Then, the sample preparation is completed.

#### 2.3.6. Comprehensive Evaluation Method

The efficacy coefficient method (ECM) has been used by many researchers to evaluate their subjects [29,30]. In this section, ECM was used to comprehensively evaluate the performance of mixed fiber-reinforced asphalt under different fiber mix ratios. According to the principle of multi-objective programming, the efficacy coefficient method requires selecting a satisfactory value as the upper limit and a disallowed value as the lower limit to calculate the average efficacy coefficient value (*F*), using Equations (2) and (3). A bigger average efficacy coefficient value (*F*) means a better comprehensive performance of MFRA. In Equations (2) and (3), $f_{i}$ is the efficacy coefficient value of a single index; $x_{i}$ is the actual test value of a single index; $x_{i}^{s}$ is the disallowed value of a single index; and $x_{i}^{h}$ is the satisfactory value of a single index.

(2) $$f_{i}=\frac{x_{i}-x_{i}^{s}}{x_{i}^{h}-x_{i}^{s}}$$

(3) $$F=\frac{\sum_{i=1}^{n}f_{i}}{n}$$

## 3. Results and Discussion

### 3.1. Softening Point

**Figure 8:**
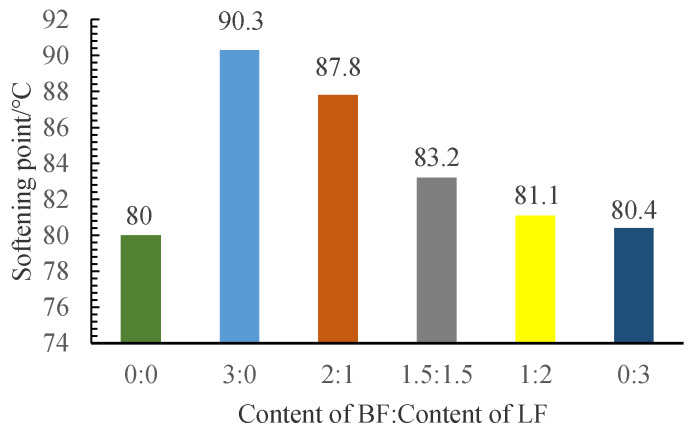
Test results of the softening point.

It can be seen from Figure 8 that the fiber mix ratio (FMR) has a great influence on the increase of the softening point. By comparison and calculation, the softening point of asphalt with 3% BF (FMR is 3:0) increased by 12.9% compared with that without fiber. In the previous analysis of the macroscopic and microscopic morphology of BF, it is clear that BF is not easy to agglomerate. Therefore, BF can disperse more evenly in the SBS asphalt and form a three-dimensional network structure [28,31], which could make the MFRA more stable and hinder the flow of asphalt to some extent and eventually make the softening point higher.

In the previous comparison of BF and LF properties, it shows that the heat resistance of BF is nearly 5 times higher than that of LF. With the increase of temperature, the property of LF in the asphalt is more unstable, which could lead to the decrease of the enhancement effect of fibers on the softening point. The softening point of asphalt with 3% LF (FMR is 0:3) increased only by 0.5% compared with that without fiber. The lower the FMR value, the worse the enhancement effect on the softening point will be. It can be concluded that BF can better improve the high-temperature performance of SBS asphalt.

### 3.2. Ductility

**Figure 9:**
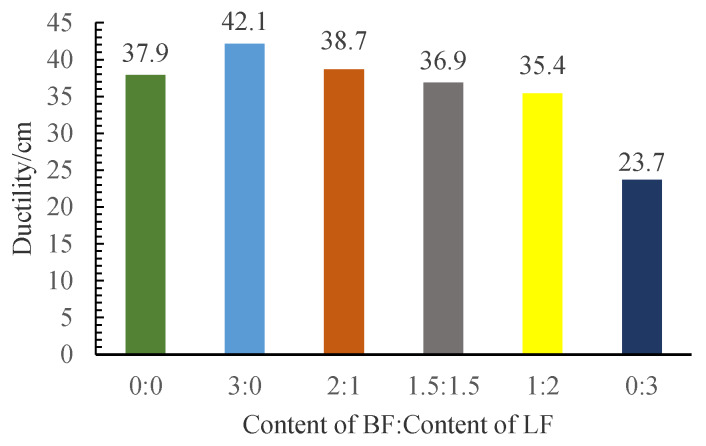
Test results of ductility.

According to Figure 9, 3% basalt fiber can significantly improve the low-temperature ductility of SBS-modified asphalt. After calculation, the ductility of it increased by 11.1% compared with the asphalt without fiber. The results are in accordance with the test results of softening point. As is shown in Table 2, the fracture strength and the elasticity modulus of BF is much better than LF. Then, BF could participate more in the test process than LF and disperse some part of the stress [28], and this could lead to the increase of ductility. This is also in accordance with the findings that BF can enhance the stiffness capacity of asphalt [31].

It can be seen that when the fibers are all LF (FMR is 0:3), the ductility of MFRA is even lower than that of SBS asphalt without fiber, and the ductility decreased by 37.5%. This phenomenon can be explained by three reasons. Firstly, the length of LF is only 13.3% of the length of BF, which could make the fibers harder to transmit the stress. Secondly, the fracture strength of LF is much smaller than BF. The total content of fibers is set as 3%, so when the content of LF is bigger, the content of BF will be smaller. This will make the fibers bear less stress in the MFRA because LF could not bear as much stress as BF does. Thirdly, LF is more likely to agglomerate in asphalt, which is consistent with the macro morphology of it, so adding LF into the asphalt might make the property of MFRA more unstable and eventually make the ductility decrease.

### 3.3. Rheological Properties

**Figure 10:**
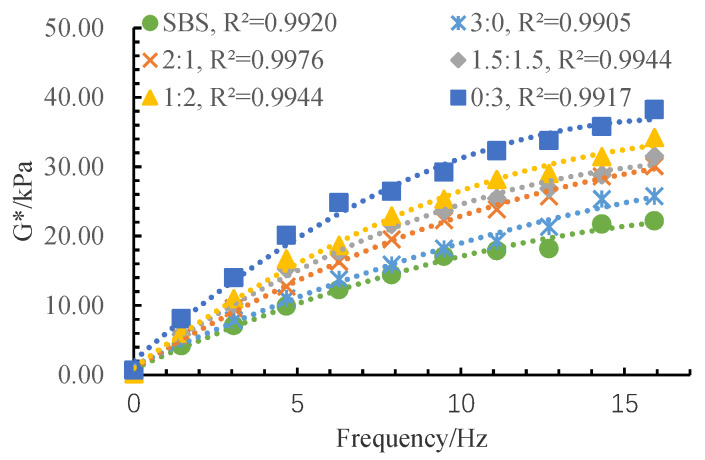
Test results of complex modulus.

**Figure 11:**
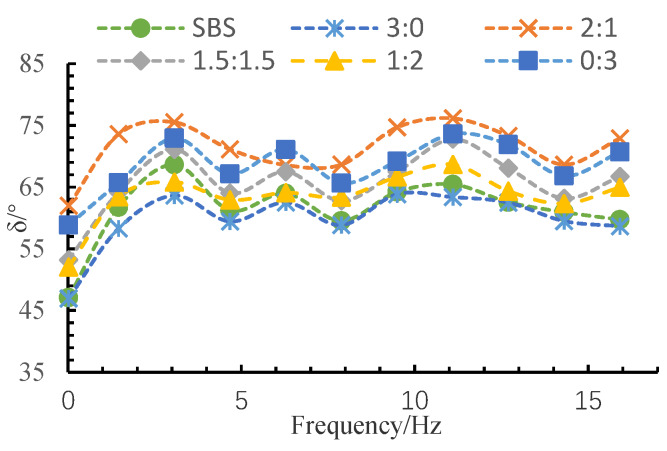
Test results of phase angle.

**Figure 12:**
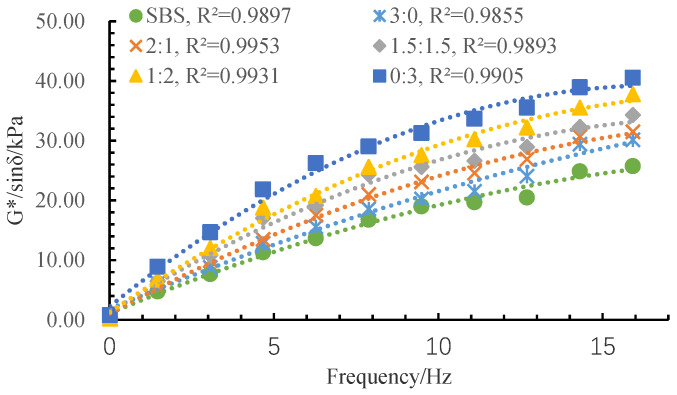
Test results of rutting factor.

The complex modulus (G*) can be divided into two parts: elasticity and viscosity. A larger G* value represents the greater stiffness and the deformation resistance of asphalt. Phase angle (δ) can stand for the time lag between the stress and the strain in the test. A lower phase angle means better elasticity. The rutting factor (G*/sin δ) can be used to access the high-temperature stability of asphalt, and a higher rutting factor implies better rutting resistance. The test results of the rheological properties of the MFRA under different fiber mix ratios are shown in Figure 10, Figure 11 and Figure 12.

It can be seen from Figure 10 that G* increases after using fibers in the SBS asphalt. With the increase of the FMR value, the complex modulus becomes lower. This means that LF could better improve the stiffness and the deformation resistance of MFRA. This phenomenon can be explained by two aspects. Firstly, Table 2 shows that the pH value of LF is about 7.04% higher than that of BF. As a result of the weak acidity of asphalt, the chemical bond ability between lignin fiber and asphalt is better than that between basalt fiber and asphalt. Secondly, the texture of LF is softer, so it is easier to blend into asphalt to make its property change and eventually enhance its stiffness and the deformation resistance. This can be ascribed by the fact that lignin fiber has a lot of polar groups, similar to the carboxyl groups and the phenolic hydroxyl groups [32], which contribute to the establishment of interactions with the asphalt molecules and their clusters, and the complex viscosity modulus is determined eventually by the force field between the molecules (the strength of the interactions involved in the intermolecular network in asphalt) [33]. As is mentioned before, BF can form a three-dimensional network structure and participate more in the softening point and ductility test process and disperse some part of the stress [28]. However, the rheology test is to test the inner property of the MFRA, so LF shows a better enhancement effect on G*.

It can also be concluded that G* is positively correlated with the loading frequency. This is because that asphalt is a kind of viscoelastic material. The decrease of the loading time will make the deformation of asphalt smaller, which will eventually lead to the increase of modulus in the test. Besides, FMR has more of an impact on the G* when the frequency is bigger, meaning that when the designed traffic volume is bigger, FMR should be paid more attention in the design of the mixed fiber-reinforced asphalt mixture.

As is shown in Figure 11, the phase angle fluctuates with the increase of frequency but the fluctuation range is limited, which is mainly because of the significant delayed effect of MFRA. Furthermore, the fluctuation phenomenon is in accordance with the test results given by Gu [4]. When the FMR is 2:1, 0:3, 1.5:1.5, 1:2, and 3:0, the phase angle of MFRA decreases in turn. When the fibers are all BF (FMR is 3:0), the phase angle is the smallest, which means that the elastic property is the best. This is mainly due to the high elastic modulus of BF. According to the mechanical principle of composite materials, the overall elasticity composite material is decided by the elasticity of its composition. So, the high elastic modulus of BF will definitely increase the elastic modulus of MFRA.

From Figure 12, the change pattern of G*/sin δ is the same as G*. LF shows a better enhancement effect on G*/sin δ than BF, and FMR has more of an impact on the G*/sin δ when the frequency is bigger. The reasons for these conclusions are the same as that of G*.

### 3.4. Oil Absorption Rate of Mixed Fibers

Table 4 presents that when the fibers are all LF (FMR is 0:3), the oil absorption rate is the biggest, and the amount of asphalt that LF could absorb 8.4 times its own weight. This is the reason why lignin fiber is widely used in the SMA asphalt mixtures to absorb asphalt. With the increase of BF, the oil absorption rate gradually gets smaller, and when the fibers are all BF (FMR is 3:0), the oil absorption rate is the smallest: just half of that of LF. This result means that the oil absorption ability of LF is much better than that of BF [28]. The specific surface area of LF is 12.87 times of that of BF. Under the same evaluation standard (the same weight), LF will have more contact area with asphalt, which will make its oil absorption rate higher. The micro image of LF shows that lignin fibers can interweave with each other, which will form a mechanical riveting structure to hold more asphalt. All these reasons contribute to the higher oil absorption rate of LF.

### 3.5. Comprehensive Performance Evaluation

In the comprehensive evaluation of MFRA using the efficacy coefficient method (ECM) [29,30], the adopted indexes in the calculation of efficacy coefficient value are the softening point, ductility, rutting factor, and oil absorption rate. The softening point represents the high-temperature performance, ductility represents the low temperature performance, the rutting factor represents the rheological properties, and the oil absorption rate represents the asphalt absorption ability of the mixed fibers. In the calculation, the disallowed value is set as the worst test value of the test value vector of a single index (such as the softening point) under different fiber mix ratios, and the satisfactory value is set as the best test value of the test value vector of a single index. It should be mentioned that the test value vector of the rutting factor is the average test value vector under all the load frequencies.

The result in Table 5 shows that when the FMR is 1:2, the comprehensive performance of MFRA is the best. However, in the design of a specific fiber-reinforced asphalt having a specific performance priority, it is necessary to determine the FMR according to the test results of that specific index (such as softening point).

### 3.6. Micromorphology of Fibers and Test Samples

A variety of composite interface theories have been put forward in studies about composite materials, and each theory has a certain experimental basis and can explain some experimental phenomena. However, due to the complexity of the mechanism of composite interface, the research studies on it are still very superficial. Therefore, in this section, scanning electron microscopy (SEM) was used to observe the micro morphology and interface structure of fiber-reinforced asphalt, and chemical bond theory, interface layer theory, friction theory, etc. [34] are adopted to analyze the strengthening mechanism of SBS asphalt reinforced by mixed fibers under different fiber mixing ratios (FMRs).

#### 3.6.1. Micro Morphology of Fibers

**Figure 13:**
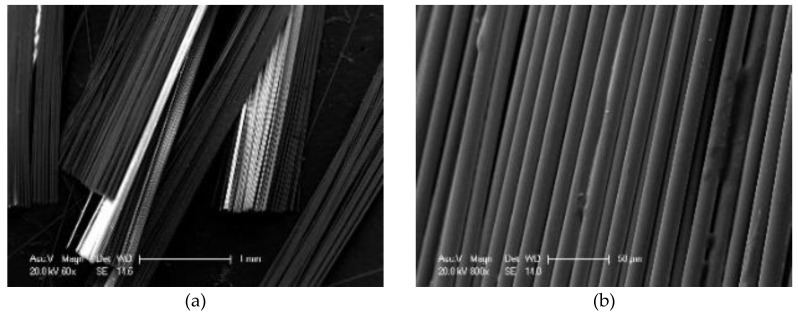
Micro image of BF: (**a**) 60×; (**b**) 800×.

The micro images of basalt fibers are shown in Figure 13. From the micro image, the shape of a single basalt fiber is similar to that of a cylinder, and it has a smooth surface. The diameter and the thickness are very uniform, which will contribute to the stability of BF. There are some little bulges on the surface of basalt fiber, which is mainly caused by the high temperature [19] during its production.

**Figure 14:**
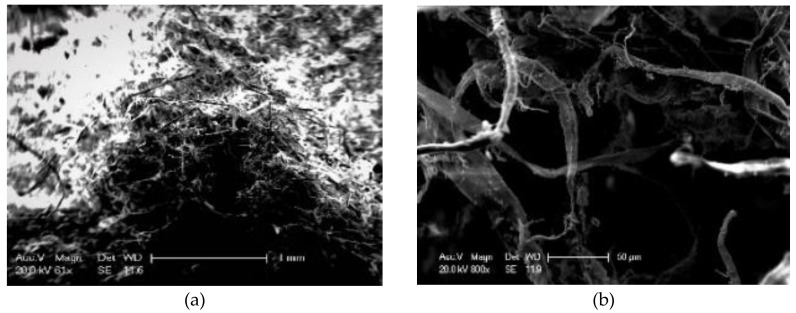
Micro image of LF: (**a**) 60×; (**b**) 800×.

The micro images of lignin fibers are shown in Figure 14. It can be seen that lignin fibers interweave with each other, which will make it hard to disperse. The shape of LF is similar to that of a curve, and the surface of it is very rough.

#### 3.6.2. Micromorphology of MFRA Samples

**Figure 15:**
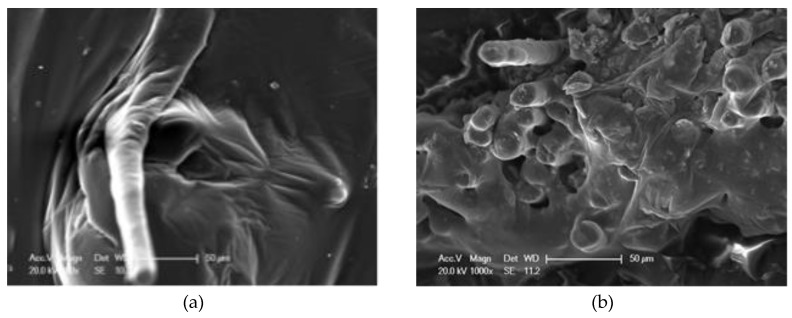
Micro images of mixed-fiber-reinforced asphalt (MFRA) (3:0): (**a**) image 1, 1000×; (**b**) image 2, 1000×.

**Figure 16:**
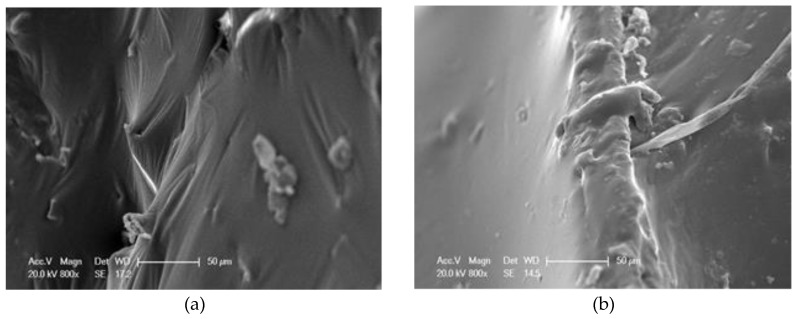
Micro images of MFRA (0:3): (**a**) image 1, 800×; (**b**) image 2, 800×.

**Figure 17:**
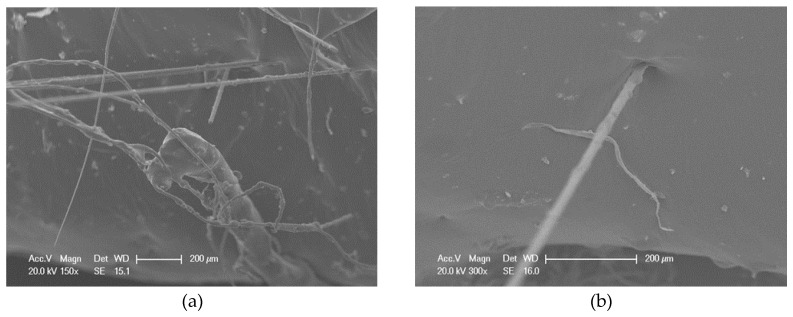
Micro images of MFRA (1.5:1.5): (**a**) image 1, 150×; (**b**) image 2, 300×.

SEM was used to observe the micro morphologies of mixed fiber-reinforced asphalts (MFRA) with three different fiber mix ratios (FMRs): 3:0, 1.5:1.5 and 0:3. The micro images of these three kinds of MFRAs are shown in Figure 15, Figure 16 and Figure 17. According to the SEM images and composite interface theories, it shows that there is an interface layer between the fiber and asphalt, and this could make the fiber bond with the asphalt better. The property of the interface layer is decided by the properties of asphalt and fiber, and it will eventually affect the performance of the fiber–asphalt composite material.

Table 2 shows that the pH value of basalt fiber is 7.1, and this means that BF is alkaline. Thus, BF could bond with asphalt with the chemical bonds because asphalt is a weak acid material [22]. The previous section shows that there are also some little bulges on the surface of basalt fiber, and this will increase the friction between BF and asphalt. It can be seen from Figure 15a that when bonding with asphalt, BF is completely wrapped by the asphalt, and the interface layer also has some bulges. According to Figure 15b, there are many basalt fibers exposed on the fracture surface of the test sample, which could contribute to the enhancement of the performance of the asphalt.

From Figure 16a, there are a lot of folds on the interface layer between LF and asphalt. This is mainly because that LF is very soft and it could curl and contact with asphalt, which will absorb more asphalt on the interface layer. According to Figure 16b and Figure 4, different shapes of lignin fibers could interweave with each other and form a mechanical riveting structure, and this structure could strengthen the properties of the asphalt.

Figure 17 shows that BF and LF scatter irregularly in the asphalt. Basalt fibers and lignin fibers could interweave with each other and lignin fibers could wind around the basalt fibers, which will form a more solid three-dimensional network structure. This structure will make the bonding ability between the mixed fibers and the asphalt better, ensure the mixed fibers hold more asphalt, and eventually improve the performance of MFRA. Apart from their respective enhancement effects on the asphalt matrix, this combined network structure formed by BF and LF can be classified as the novel or emerging properties of the MFRA compared with the single-fiber reinforced asphalt binder. Furthermore, the functional groups of BF and LF might make this structure more stable, because adding different additives might form the amphiphilic molecules (from the microscopic view), which will trigger the emerging properties or functions [35]. According to Figure 17b, BF is much longer than LF, and BF is always straight. This is mainly because that the elasticity modulus of BF is very high, and the texture of BF is very hard. This characteristic will make the strength and the elasticity of the MFRA better.

#### 3.6.3. The Failure Model of Fiber-Reinforced Asphalt

**Figure 18:**
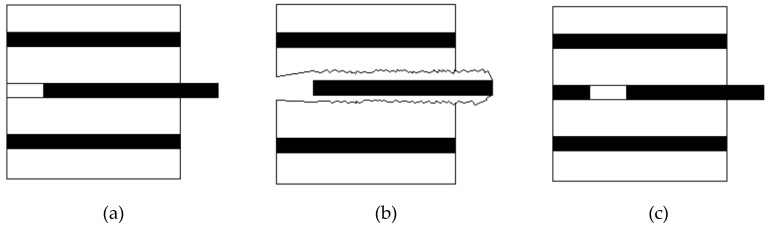
Failure situations: (**a**) fiber slips out from asphalt; (**b**) fiber wrapped with asphalt in the pull-out process; (**c**) fiber breaks in the asphalt.

The composition of fiber-reinforced asphalt is fiber and asphalt binder. When they mix with each other, they will bond together due to the action of molecular forces and chemical bonds. The mechanisms of these actions are very complex, but they will contribute to the strengthening of the asphalt [36]. The failure model of the fiber-reinforced asphalt could be summarized into three typical situations, which are shown in Figure 18. Figure 18a shows the failure situation that the fiber-reinforced asphalt is broken due to the slide of the fiber from the asphalt, which is mainly because that the bonding between the fiber and the asphalt is not strong enough to hold the pull-out force. This mode can be classified as the interface failure mode [37]. Figure 18b shows that the fiber is wrapped by the asphalt and pulled out along with some of the asphalt, and this can be viewed as the matrix failure mode [37]. In this situation, the bonding between the fiber and the asphalt is strong enough to hold the pull-out force. The failure situation shown in Figure 18c presents that the fiber is fractured when it is being pulled out from the asphalt, and this is mainly because the fiber could bond well with asphalt, but the pull-out force is bigger than the fracture strength of the fiber itself. This phenomenon can be ascribed to the weak strength of the fiber or the strong adhesion between the fiber and the asphalt matrix. Generally, it could be concluded that the failure model of the fiber-reinforced asphalt will be affected by the bonding situations between the fiber and the asphalt and the fracture strength of the fiber itself.

## 4. Conclusions

After conducting a series of studies on the performance and strengthening mechanism of mixed fiber-reinforced asphalt with different fiber mix ratios, the following conclusions can be drawn:

1. Basalt fiber improves the softening point and ductility of MFRA better than lignin fiber. A lower FMR value will cause a worse enhancement effect on the softening point and the ductility. Lignin fiber increases the complex modulus (G*) and the rutting factor (G*/sin δ) of MFRA more significantly than basalt fiber. FMR has more impact on the G* and G*/sin δ in higher frequency. The phase angle fluctuates with the increase of frequency, and the fluctuation range is limited.
2. Lignin fiber absorbs more asphalt than basalt fiber. The amount of asphalt that lignin fiber could absorb is 8.4 times its own weight. With the increase of basalt fiber, the oil absorption rate of the fiber mix gradually gets smaller and it is just half of that of LF when the fibers are all BF (FMR is 3:0).
3. The FMR is proposed as 1:2 according to the analysis by the efficacy coefficient method. The satisfactory comprehensive performance of MFRA is available when the FMR is 1:2.
4. It was observed from the SEM images that basalt fiber and lignin fiber interweaved with each other and lignin fiber wound around the basalt fibers, which formed a more stable three-dimensional network structure to make the mixed fibers hold more asphalt and eventually improve the performance of MFRA.

## Figures and Tables

**Table 1:** Properties of styrene–butadiene–styrene (SBS) modified asphalt.

Index	Value
Penetration (25 °C, 100 g, 5 s), 0.1 mm	59.5
Softening point (TR&B), °C	80.0
Ductility (5 °C, 5 cm/min), cm	37.9
Solubility, %	99.9
Elastic recovery (25 °C), %	98.7
Rotational viscosity (135 °C), Pa·s	2.302
Relative density (25 °C)	1.031

**Table 2:** Properties of basalt fiber and lignin fiber.

Index	Basalt Fiber	Lignin Fiber
Length, mm	6	0.8 (Average)
Diameter, μm	14	8
Length–diameter ratio	428.6	100
Density, g/cm^3^	2.710	0.910
Specific surface area, m^2^/g	0.15	1.93
Hygroscopic rate, %	1.63	28.70
Heat resistance, ℃	1550	260
PH value	7.1	7.6
Fracture strength, MPa	≥2000	<300
Modulus of elasticity, GPa	100	30

**Table 3:** Fiber mix ratio. BF: basalt fiber, LF: lignin fiber.

BF:LF	3:0	2:1	1.5:1.5	1:2	0:3

**Table 4:** Results of the basket leak tests. *OA*: (oil) absorption rate.

BF:CF	3:0		2:1		1.5:1.5		1:2		0:3	
*m*_1_, g	9	9	9	9	9	9	9	9	9	9
*m*_2_, g	199.8	200	199.6	200	200	201	200.2	200	200	199.9
*m*_3_, g	247.5	245.9	259.9	260.3	265.7	270.3	274	275.6	282.8	286.3
*OA*	4.3	4.1	5.7	5.7	6.3	6.7	7.2	7.4	8.2	8.6
Average	4.2		5.7		6.5		7.3		8.4	

**Table 5:** Calculation of the efficacy coefficient.

Index	Satisfactory Value	Disallowed Value	BF:LF				
			3:0	2:1	1.5:1.5	1:2	0:3
Softening point/°C	90.3	78.4	90.3	87.9	83.2	81.1	78.4
			1.0000	0.7983	0.4034	0.2269	0.0000
Ductility/cm	42.1	23.9	42.1	38.7	36.9	35.4	23.7
			1.0000	0.8152	0.7174	0.6359	0.0000
Rutting factor/kPa	25.58	17.07	17.07	18.61	20.56	22.50	25.58
			0.0000	0.1812	0.4106	0.6385	1.0000
Oil absorption rate	8.4	4.2	4.2	5.7	6.5	7.3	8.4
			0.0000	0.3571	0.5476	0.7381	1.0000
Average efficacy coefficient value			0.5000	0.5380	0.5197	0.5598	0.5000
